# Supplementary material for: The acceptability of minimally invasive tissue sampling for cause of death determination in rural South Africa: A qualitative analysis
Source: PLoS One. 2026 May 22;21(5):e0345107. doi: 10.1371/journal.pone.0345107 (PMC13196941; doi:10.1371/journal.pone.0345107)
Supplement: Attachment — Submitted filename: Response to reviewers.docx [file pone.0345107.s003.docx]

03/11/2025

Dear Editors,

**RE: MANUSCRIPT NO: PONE-D-24-56472.Title: “The acceptability of minimally invasive tissue sampling for cause of death determination in rural South Africa: a qualitative analysis”**

I’d like to thank the reviewers of PLOSOne for their feedback on the manuscript titled “The acceptability of minimally invasive tissue sampling for cause of death determination in rural South Africa: a qualitative analysis”. I appreciate the comments and am happy to provide clarification on any points that were unclear.

I report changes made to satisfy the journal’s formatting and information requirements at the end of this letter.

I have responded to each reviewer’s query in the table provided below and included where any changes will be reflected in the updated manuscript. Changes made to the original manuscript are reflected in the document labelled “Revised Manuscript with Tracked Changes”.

| **Reviewer comments** | **Author response** | **Line/page number** | **Changes** |
| --- | --- | --- | --- |
| For a scientific study which will be helpful to the community, why so remote area is selected? | This study was purposefully set in a rural community: firstly, in rural areas there are more gaps in cause of death data as resources to determine a clinical cause of death are more limited and a larger proportion of the population die outside of health facilities. Alternative autopsy methods, like MITS, could play an important role in filling that gap.  Secondly, the structural and cultural barriers to conducting MITS are expected to be different in urban and rural settings. While there is already a well-established MITS project in SOWETO, - a peri-urban setting outside of Johannesburg in South Africa, no work has been done in rural areas in the region. This paper will specifically look at the acceptability of MITS for community deaths within a rural setting. | Covered in introduction (Line 82–85) | Included in updated manuscript as follows: *“However, the majority of MITS work has been restricted to facility-based deaths, deaths in children under 5 and stillbirths (17–24). There is a need to understand the acceptability of MITS in community deaths across all ages in a rural setting, in order to inform the implementation of* *MITS in out-of-hospital, resource-limited settings.”* |
| The study is based only on the basis of question and answer by interview, or any follow up study | This study utilised focus group discussion and in-depth interviews only. A follow up study is underway, but results are not yet available and did not contribute to the findings of this manuscript. | Line 101 -102(Methods) | Included in the updated manuscript as follows: *“This qualitative study consisted of in-depth interviews (IDIs) and focus groups discussions (FGDs) with community members and key stakeholders.”* |
| Line 28-29 experience of death in family, How it will help in study? does this include the experience of cause of death investigation and any disgusting feeling by them during that procedure. | Participants who had recently experienced death would have more insight into the emotions and ritual practices surrounding death in this context. They will be able to give more in-depth and personal accounts of their experience and offer insight into how they may have felt if we had approached them at the time of death to participate in MITS. The vast majority of community members have never witnessed a formal autopsy, so this was not part of our inclusion criteria, although we did ask about their opinions on post-mortem investigations. We also showed participants the MITS biopsy needles, and allowed them to handle the needle including hearing the clicking sound made by the punch-biopsy needles, to gauge their responses. We felt that showing them the needles would help them better understand what would be involved in a MITS, as opposed to simply describing the procedure in theory, or showing photographs of the needles. | Methods – Sampling (135-137) | Included in the updated manuscript as follows: *“The Agincourt HDSS database was used to identify individuals who had experienced a death in the 2 years prior; participants could draw on the experience of a recent loss to formulate responses.”* |
| Does inform consent was in camera? there is no clarification. | All interviews and focus group discussions were audio-recorded only, no video-recording was used. Similarly, consent was taken in writing and as part of the audio-recording. The informed consent process included specific consent to participate in the interview and separately to be audio-recorded. | Line 157-158 | Included in updated manuscript as follows:  *“For the IDIs, two interviewers approached potential participants at their homes to obtain consent to conduct and audio-record the interview. ”* |
| There is no clarification about the type of tissue and from which site of body samples will be collected. | Our apologies for the lack of clarification in this regard: this study was an explorative study prior to the MITS intervention study, designed to better understand factors that may affect the acceptability of MITS, so that the study design and implementation could be adapted to respect the wishes of the community. However, the basics of the MITS methodology are standardised across all study sites, as outlined in the RTI alliance MITS protocol (referenced in the update manuscript). We have also included a brief description of the MITS methodology in the methods section. | Line 103-108 | Added to the updated manuscript as follows: *“This study is part of formative research conducted to better inform the implementation of MITS this specific context. The proposed study will aim to conduct MITS on deaths occurring outside of healthcare facilities, collecting samples of brain, lung, liver, heart, kidney and splenic tissue, in addition to blood and cerebrospinal fluid to determine cause of death (27, 28).”* |
| Who will collect the samples and who will process them? | We have not included this level of detail in the methodology as this study is not about the MITS intervention study itself, but rather a qualitative study undertaken as part of the planning process to optimise efforts to introduce MITS into the community and ensure high participation and acceptance rates. |  |  |
| What will be the cost effectiveness of such investigations? | No cost-effectiveness studies of MITS have been conducted to date, and this was beyond the scope of this study. |  |  |
| If the community agree on the study, does author has proceeded for sampling and if yes what was the results?  Is the technique is applicable only to nature undiagnosed deaths or it can be used for unnatural deaths to know cause of death? Have author conducted any such study by taking minimum tissues? If yes what was the result? | After this qualitative study was conducted, the research team obtained permission from all relevant authorities (including community leaders) to proceed with MITS. The MITS study is still ongoing, and the results will be published separately from this paper. |  |  |
| Is the technique is applicable only to nature undiagnosed deaths or it can be used for unnatural deaths to know cause of death? Have author conducted any such study by taking minimum tissues? If yes what was the result? | MITS is a relatively novel technique and its application in unnatural deaths has not been investigated before.  This paper is part of formative work conducted before MITS was implemented in the Agincourt HDSS. The main study is still ongoing, and no results are available yet. |  |  |
| What is the total population of study area? | The latest estimates include around 117 000 within the Agincourt Health and socio-Demographic Surveillance System platform. | Line 116-117 | Included in the updated manuscript as follows: *“The HDSS covers 31 villages and a population of some 117,000 people.”* |
| Introduction: Line 53 “ a useable” – can be replaced with a better word |  | Line 54 | Replaced:  *“a useable”*  With  *“an informative”* |
| Results: 197 Table 3. Summary of FGD participants – Table incomplete? | Our apologies if there was an error in uploading table 3. We have double checked that the table is not missing any information and have uploaded a new copy. Please confirm that the complete table is uploaded (there should be no blank cells). |  |  |

Thank you for considering this manuscript and giving me the opportunity to respond to your queries. I look forward to your response.

Sincerely,

Laura-Lynne Brandt, DPhil candidate

SAMRC/Wits Rural Public Health and Health Transitions Research Unit (Agincourt) University of the Witwatersrand

[2207756@students.wits.ac.za](mailto:2207756@students.wits.ac.za)
