# Supplementary material for: The acceptability of minimally invasive tissue sampling for cause of death determination in rural South Africa: A qualitative analysis
Source: PLoS One. 2026 May 22;21(5):e0345107. doi: 10.1371/journal.pone.0345107 (PMC13196941; doi:10.1371/journal.pone.0345107)
Supplement: Attachment — Submitted filename: Response_to_reviewers_auresp_2.docx [file pone.0345107.s004.docx]

18/12/2025

Dear Reviewers,

**RE: MANUSCRIPT NO: PONE-D-24-56472.Title: “The acceptability of minimally invasive tissue sampling for cause of death determination in rural South Africa: a qualitative analysis”**

We would like to thank the reviewers for considering our updated manuscript titled “The acceptability of minimally invasive tissue sampling for cause of death determination in rural South Africa: a qualitative analysis”. We understand that there are still some points that you found to be unclear and we are happy to provide further explanation.

We have responded to each reviewer’s query in the table provided below and included where any changes will be reflected in the updated manuscript.

| **Reviewer comments** | **Author response** |
| --- | --- |
| Still the point is not clear to me, why the author chose to study on the interview basis? | Previous studies exploring the acceptability of MITS also utilised interviews to collect their data. Our rationale for using interviews and focus group discussions to explore this topic was to get a detailed account of factors affecting the acceptability of MITS and to explore other practical aspects around the implementation of MITS in the Agincourt HDSS area. Quantitative methods would not have allowed us to get the depth of information we needed to inform the implementation of MITS in this setting. I have included references to other studies that have explored the acceptability of MITS using qualitative interviews after this table. These studies have also been cited in the manuscript. |
| Why general public was involved in a study which require authorization from higher authorities? | This study was approved by the University of the Witwatersrand Human Research Ethics Committee (Medical) and from the Mpumalanga Provincial Health Research and Ethics Committee as these were the local ethics committees responsible for enforcing ethical standards in research in the area. This was not a national project, but a research study conducted only within the Agincourt HDSS area. |
| The author has not discussed about cost effectiveness of the test because if it is free burdone to goventment and if chargeble then people have to know the cost per test. | This study is purely for research purposes, and all the costs of the project are covered by research grants. This is not a service that is provided by the South African government currently nor a service that is available to the general community on request at their own cost. For this study there is no cost to the consumer or to the government. A cost effectiveness analysis can be conducted at a later stage once full costing implications are better understood. The research presented in this manuscript was a qualitative investigation into the acceptability of MITS in a rural community and so costings were not reflective of the MITS procedure itself |

Articles investigating the acceptability of MITS:

1. Magaço A, Alonso Y, Maixenchs M, Ambrosio C, Sitoe A, Vitorino P, et al. A Qualitative Assessment of Community Acceptability and Its Determinants in the Implementation of Minimally Invasive Tissue Sampling in Children in Quelimane City, Mozambique. Am J Trop Med Hyg. 2023 Apr 10;tpmd220343.
2. Ngwenya N, Coplan D, Nzenze S, Myburgh N, Madhi S. Community acceptability of minimally invasive autopsy (MIA) in children under five years of age in Soweto, South Africa. Anthropol South Afr. 2017 June 2;40(2):108–21.
3. O’Mara Sage E, Munguambe KR, Blevins J, Guilaze R, Kosia B, Maixenchs M, et al. Investigating the Feasibility of Child Mortality Surveillance With Postmortem Tissue Sampling: Generating Constructs and Variables to Strengthen Validity and Reliability in Qualitative Research. Clin Infect Dis Off Publ Infect Dis Soc Am. 2019 Oct 9;69(Suppl 4):S291–301.
4. Das MK, Arora NK, Kaur G, Malik P, Kumari M, Joshi S, et al. Perceptions of family, community and religious leaders and acceptability for minimal invasive tissue sampling to identify the cause of death in under-five deaths and stillbirths in North India: a qualitative study. Reprod Health. 2021 Aug 4;18(1):168.
5. Feroz A, Ibrahim MN, McClure EM, Ali AS, Tikmani SS, Reza S, et al. Perceptions of parents and religious leaders regarding minimal invasive tissue sampling to identify the cause of death in stillbirths and neonates: Results from a qualitative study. Reprod Health [Internet]. 2019;16(1). Available from: https://www.scopus.com/inward/record.uri?eid=2-s2.0-85065569015&doi=10.1186%2fs12978-019-0730-9&partnerID=40&md5=330380b6971fd968805251084a64f4ed
6. Lawrence S, Namusanya D, Hamuza A, Huwa C, Chasweka D, Kelley M, et al. Hypothetical acceptability of hospital-based post-mortem pediatric minimally invasive tissue sampling in Malawi: The role of complex social relationships. PloS One. 2021;16(2):e0246369.
7. Rugwizangoga B, Niyibizi JB, Ndayisaba MC, Musoni E, Manirakiza F, Uwineza A, et al. Exploring Perceptions and Acceptance of Minimally Invasive Tissue Sampling among Bereaved Relatives and Health-Care Professionals in Rwanda. J Multidiscip Healthc. 2021 Dec 15;14:3421–7.
8. Ahsan N, Malik FA, Mughis W, Jabeen R, Mehboob S, Allana R, et al. Postmortem minimally invasive tissue sampling in communities: exploring perceptions of families, funeral workers, religious and community leaders and healthcare providers from Pakistan. BMC Health Serv Res. 2023 Dec 13;23(1):1402.

Thank you for considering this manuscript and giving me the opportunity to respond to your queries. I look forward to your response.

Sincerely,

Laura-Lynne Brandt, DPhil candidate

SAMRC/Wits Rural Public Health and Health Transitions Research Unit (Agincourt) University of the Witwatersrand

[2207756@students.wits.ac.za](mailto:2207756@students.wits.ac.za)
