# Supplementary material for: The acceptability of minimally invasive tissue sampling for cause of death determination in rural South Africa: A qualitative analysis
Source: PLoS One. 2026 May 22;21(5):e0345107. doi: 10.1371/journal.pone.0345107 (PMC13196941; doi:10.1371/journal.pone.0345107)
Supplement: Attachment — Submitted filename: Reviewer comment.docx [file pone.0345107.s001.docx]

The authors address the issue of acceptability of MITS for determining the cause of death in rural South Africa

The paper is well written and clear.

Recommendations: Accept

My Comments:

Introduction: Line 53 “ a useable” – can be replaced with a better word

Methodology: No change

Results: 197 Table 3. Summary of FGD participants – Table incomplete?

Conclusions: No change
